# *Rhodiolae Kirliowii Radix et Rhizoma* and* Crataegus pinnatifida Fructus* Extracts Effectively Inhibit BK Virus and JC Virus Infection of Host Cells

**DOI:** 10.1155/2017/5620867

**Published:** 2017-07-03

**Authors:** San-Yuan Chen, Ru-Hsiou Teng, Meilin Wang, Pei-Lain Chen, Mien-Chun Lin, Cheng-Huang Shen, Chun-Nun Chao, Ming-Ko Chiang, Chiung-Yao Fang, Deching Chang

**Affiliations:** ^1^Department of Chinese Medicine, Ditmanson Medical Foundation Chiayi Christian Hospital, Chiayi, Taiwan; ^2^Institute of Molecular Biology, National Chung Cheng University, Chiayi, Taiwan; ^3^Department of Microbiology and Immunology, Chung Shan Medical University, Taichung, Taiwan; ^4^Department of Medical Laboratory Science and Biotechnology, Central Taiwan University of Science and Technology, Taichung, Taiwan; ^5^Department of Urology, Ditmanson Medical Foundation Chiayi Christian Hospital, Chiayi, Taiwan; ^6^Department of Pediatrics, Ditmanson Medical Foundation Chiayi Christian Hospital, Chiayi, Taiwan; ^7^Department of Medical Research, Ditmanson Medical Foundation Chiayi Christian Hospital, Chiayi, Taiwan

## Abstract

The human polyomaviruses BK (BKPyV) and JC (JCPyV) are ubiquitous pathogens long associated with severe disease in immunocompromised individuals. BKPyV causes polyomavirus-associated nephropathy and hemorrhagic cystitis, whereas JCPyV is the causative agent of the fatal demyelinating disease progressive multifocal leukoencephalopathy. No effective therapies targeting these viruses are currently available. The goal of this study was to identify Chinese medicinal herbs with antiviral activity against BKPyV and JCPyV. We screened extracts of Chinese medicinal herbs for the ability to inhibit hemagglutination by BKPyV and JCPyV virus-like particles (VLPs) and the ability to inhibit BKPyV and JCPyV binding and infection of host cells. Two of the 40 herbal extracts screened,* Rhodiolae Kirliowii Radix et Rhizoma *and* Crataegus pinnatifida Fructus*, had hemagglutination inhibition activity on BKPyV and JCPyV VLPs and further inhibited infection of the cells by BKPyV and JCPyV, as evidenced by reduced expression of viral proteins in BKPyV-infected and JCPyV-infected cells after treatment with* Rhodiolae Kirliowii Radix et Rhizoma *or* Crataegus pinnatifida Fructus *extract. The results in this work show that both* Rhodiolae Kirliowii Radix et Rhizoma* and* Crataegus pinnatifida Fructus* may be sources of potential antiviral compounds for treating BKPyV and JCPyV infections.

## 1. Introduction

The human BK polyomavirus (BKPyV) and JC polyomavirus (JCPyV) are ubiquitous pathogens [1]. Infection by these human polyomaviruses usually takes place in childhood and is asymptomatic, followed by latency in the kidney and the urinary tract [2]. In immunocompromised individuals, BKPyV and JCPyV can reactivate and cause severe disease. In the case of organ transplant recipients on an immunosuppressive regimen, BKPyV reactivation and replication in the renal tubular epithelium lead to polyomavirus-associated nephropathy (PVAN) in about 10% of renal transplant patients and hemorrhagic cystitis in bone marrow transplant patients. Approximately 50% of patients with PVAN ultimately progress to renal failure, leaving them in need of another kidney transplant [2]. In about 3%–5% of HIV/AIDS patients and in multiple sclerosis patients undergoing immunomodulatory therapy, the immunosuppressed state triggers reactivation of latent JCPyV, whose lytic infection of oligodendrocytes results in the fatal demyelinating disease progressive multifocal leukoencephalopathy (PML). Death usually occurs 1-2 years after PML diagnosis [3].

Recent advances in diagnostic technology have permitted rapid quantification of BKPyV copy numbers in patients, allowing the dosage of immunosuppressants to be lowered when high levels of BKPyV replication are detected. However, reduction of immunosuppression as a strategy to limit BKPyV replication in renal transplant patients was not correlated with significantly decreased rates of renal graft loss due to PVAN and carries a risk of graft rejection [2]. Treatment of PML caused by JCPyV reactivation remains focused on reconstituting the patient's immune system. Combined antiretroviral therapy is the usual treatment for AIDS patients with detectable JCPyV in the cerebrospinal fluid and can reduce the rate of JCPyV reactivation, but the two-year mortality rate of PML patients remained high at 50%–60% [4]. In a subset of PML patients, their immune system becomes overly activated by immune reconstitution and produces an inflammatory response to tissues affected by JCPyV reactivation, leading to PML-immune reconstitution inflammatory syndrome (PML-IRIS); such cases could necessitate measures to lower the patients' immune activity [5, 6]. Also, PML that develops in patients with hematological malignancy can be difficult to treat because chemotherapy interferes with the production of immune cells needed for the immune reconstitution strategy against JCPyV [7]. For these reasons, bolstering patients' immune function is of limited usefulness in combating reactivated BKPyV and JCPyV. A number of drugs that are not specific anti-BKPyV or anti-JCPyV agents are currently in clinical use for BKPyV or JCPyV infection. Among them, cidofovir, leflunomide, fluoroquinolones, and intravenous immunoglobulins are commonly used to treat PVAN due to BKPyV reactivation; however, these drugs have degrees of hepatic, renal, or cardiac toxicity [8–10]. Cidofovir and leflunomide have been used to treat JCPyV reactivation, but reports are scant and clinical outcomes varied [7]. Therefore, the development of effective anti-BKPyV and anti-JCPyV drugs is sorely needed.

The binding of a virus to its host cell's receptor determines tropism and tissue-specific pathology; thus, blocking this binding is a promising approach for drug development. The cell surface receptors for BKPyV are disialic acid gangliosides GD1b, GT1b, GD3, and GD2 [11]. JCPyV recognizes the complex consisting of a serotonin receptor and the sialic acid-containing pentasaccharide LSTc [12, 13]. With the increasing use of medicinal herbs in the development of antiviral drugs, the extracts of many Chinese medicinal herbs have been found to inhibit the progression of the viral life cycle at the stages of entry, replication, assembly, and release, as well as targeting specific virus-host interactions [14]. In this study, we screened the extracts of approximately 40 Chinese medicinal herbs, among which we found* Rhodiolae Kirliowii Radix et Rhizoma *and* Crataegus Pinnatifidae Fructus *extracts to effectively inhibit the hemagglutination activity of BKPyV and JCPyV virus-like particles (VLPs). The two herbal extracts were further examined for their ability to inhibit BKPyV and JCPyV infection of human kidney and neuroglial cells, respectively, and the presumed mechanism by which* Rhodiolae Kirliowii Radix et Rhizoma* and* Crataegus pinnatifida Fructus* inhibit BKPyV and JCPyV infection was also verified.

## 2. Materials and Methods

### 2.1. Purification of VLPs from Yeast Expressing BKPyV and JCPyV VP1s

Expression of VLPs from yeast and their purification has been previously described in detail [15]. The plasmids pFXBKV1 and pFXJCV1, bearing the VP1 genes of BKPyV and JCPyV, respectively, were transfected into* Saccharomyces cerevisiae *(INVSC1 yeast cells). Galactose was added to the cultures to a final concentration of 3% to induce the expression of the VP1 proteins. The yeast cells were harvest and lysed, and the supernatants of the VP1-containing lysates were subjected to centrifugation through a 20% sucrose cushion and then through a 10%–30% sucrose gradient. Fractions with hemagglutination activity were collected and concentrated with Centricon filters (Millipore, Billerica, MA).

### 2.2. Preparation of Herbal Extracts

Herbal power was purchased from Chuang Song Zong Pharmaceutical Co., Ltd. (Kaohsiung, Taiwan), and KO DA Pharmaceutical Co., Ltd. (Taoyuan, Taiwan). Herbal power was diluted in phosphate-buffered saline (PBS) to a final concentration of 100 mg/mL. After centrifugation at 14,000 rpm for 30 min, the supernatant was filtered through 0.45 *μ*m and 0.22 *μ*m filter paper and stored at −20°C until use.

### 2.3. Hemagglutination Inhibition Assay

Before being analyzed for hemagglutination inhibition activity on BKPyV and JCPyV VLPs, herbal extracts were first tested to ensure their lack of hemagglutination activity. Fifty microliters of herbal extract (100 mg/mL) was diluted in twofold steps with PBS in a U-shaped microtiter plate, to which 50 *μ*L of 0.7% (v/v) human type O erythrocyte suspension was then added. After 1 h of incubation, herbal extracts that did not exhibit observable hemagglutination activity were assayed for the ability to inhibit hemagglutination by BKPyV or JCPyV VLPs. The hemagglutination inhibition assay was performed as the previous report with a minor modification [16]. Briefly, 25 *μ*L of herbal extract was diluted in twofold steps with PBS in a U-shaped microtiter plate, to which 2 hemagglutination units (HAU) of BKPyV VLPs or 8 HAU of JCPyV VLPs in 25 *μ*L were added per well, followed by 50 *μ*L of 0.7% (v/v) human type O erythrocyte suspension. After 1 h of incubation, the hemagglutination inhibition activity of each herbal extract was defined as the highest dilution that resulted in complete inhibition of VLPs' hemagglutination activity.

### 2.4. Cells and Virus Propagation and Purification

HK-2 cells (Cat. number 60097; BCRC, Hsinchu, Taiwan) were grown in a humidified CO_2_ incubator at 37°C in 1 : 1 Dulbecco's modified Eagle's medium and Ham's F-12 nutrient mix (Invitrogen, Carlsbad, CA, USA) supplemented with 5% fetal bovine serum (Invitrogen), 5 *μ*g/mL transferrin (Millipore), 5 *μ*g/mL insulin (Sigma-Aldrich, St. Louis, MO, USA), 400 ng/mL hydrocortisone (Sigma-Aldrich), and 5 ng/mL sodium selenite (Sigma-Aldrich). SVG-A cells, a gift from Dr. W. Atwood (Brown University, RI, USA), were maintained in minimal essential medium supplemented with 10% fetal bovine serum, 1% penicillin, and 1% streptomycin. The methods of propagating and purifying BKPyV and JCPyV were similar to those described previously [17]. Briefly, 320 HAU of either the UT strain of BKPyV or the Mad-4 strain of JCPyV (purified from JCI cells) [18] were propagated in HK-2 or SVG-A cells, respectively, with a change of culture medium every other week. When the cytopathic effect was evident, virus-infected cells were harvested and suspended in buffer A (10 mM Tris-HCl, 50 mM NaCl, and 0.1 mM CaCl_2_) and lysed through three cycles of freezing and thawing at −80°C and 37°C. Viral particles in the lysate were released from cellular membranes by treatment with type V neuraminidase (Sigma-Aldrich) and then subjected to 20% sucrose cushion centrifugation and five-step CsCl gradient centrifugation. Fractions corresponding to DNA-containing virions were collected and concentrated with a Centricon filter (Millipore).

### 2.5. Cell Viability Assay

To determine the viability of cells in the presence of herbal extracts, the Cell Counting Kit 8 (CCK-8) proliferation assay (Sigma-Aldrich) and trypan blue exclusion test were used. HK-2 or SVG-A cells were seeded in 96-well plates at 1 × 10^4^ cells per well and incubated overnight. The following day,* Rhodiolae Kirliowii Radix et Rhizoma* or* Crataegus pinnatifida Fructus* extract was added to each well at various concentrations, and the cells were incubated for 72 h at 37°C in a humidified incubator. For CCK-8 assay, viability was assayed according to the CCK-8 manufacturer's instructions, and absorbance values were read at 450 nm 2 h after the addition of assay solution. For trypan blue exclusion test, quantification of viable cells was performed as the previous report [19]. Values were normalized to a vehicle-treated control, and three independent experiments were performed and used to calculate standard deviations.

### 2.6. Virus Labeling

BKPyV and JCPyV were labeled with Alexa Fluor 488 according to the manufacturer's instructions (Invitrogen). Purified BKPyV or JCPyV at 1 mg/mL was dialyzed in 0.1 M carbonate–bicarbonate buffer, pH 8.0, and dye was added at a molar excess of 200 : 1. After 1 h of rocking incubation at room temperature, excess dye was removed by centrifugation using a 30,000 MWCO Centricon filter (Millipore).

### 2.7. Flow Cytometric Scoring of Virus Binding

The effect of herbal extracts on virus binding to cells was assayed by flow cytometry. Cells were detached from plates, washed with ice-cold PBS, and resuspended in culture medium containing a selected concentration of* Rhodiolae Kirliowii Radix et Rhizoma* or* Crataegus pinnatifida Fructus *extract and Alexa Fluor 488-labeled BKPyV or Alexa Fluor 488-labeled JCPyV. The mixtures were incubated on ice for 1 h and then fixed by adding 200 *μ*L of BD Cytofix, followed by washing in ice-cold PBS three times. Binding was assessed by flow cytometry.

### 2.8. Examination of Virus Binding by Fluorescence Microscopy

Alexa Fluor 488-labeled virus, either BKPyV or JCPyV, was preincubated with* Rhodiolae Kirliowii Radix et Rhizoma* or* Crataegus pinnatifida Fructus* extract or with vehicle control for 30 min at 4°C. These mixtures were added to prechilled cells on coverslips and allowed to bind for 1 h at 4°C. The cells were washed three times in ice-cold PBS and then fixed by adding 200 *μ*L of BD Cytofix and incubating for 20 min. The coverslips were washed three times in ice-cold PBS, mounted with antifade fluorescence mounting medium (Sigma-Aldrich), and examined under a fluorescent microscope (Olympus) to visualize the effect of* Rhodiolae Kirliowii Radix et Rhizoma *or* Crataegus pinnatifida Fructus *treatment on BKPyV and JCPyV binding to host cells.

### 2.9. Analysis of Inhibition of BKPyV and JCPyV Infection by Herbal Extracts

HK-2 or SVG-A cells were seeded onto 24-well plates at 1 × 10^5^ cells per well. In the next day, the cells were pretreated with* Rhodiolae Kirliowii Radix et Rhizoma *or* Crataegus pinnatifida Fructus *extract for 30 min, and then 320 HAU of BKPyV or JCPyV were applied to the cells in 0.08 mL in the presence of* Rhodiolae Kirliowii Radix et Rhizoma* or* Crataegus pinnatifida Fructus *extract at a selected concentration. After virion adsorption was allowed to proceed for 1 h, the cells were washed with ice-cold PBS to remove the herbal extract and free virus and overlaid with 0.5 mL of culture medium per well. After 72 h of incubation, the percentage of virus infection was scored by immunofluorescence assay and quantitative real-time PCR.

### 2.10. Immunofluorescence Assay

Herbal extract- or vehicle-treated, virus-infected cells on coverslips were fixed with cold acetone and methanol. The coverslips were blocked with normal horse serum (Invitrogen), and the cells' morphology was revealed by counterstaining with Evan's blue solution. The cells were then incubated with mouse monoclonal antibody against simian virus 40 (SV40) large T antigen (LT) (Calbiochem), which cross-reacts with BKPyV LT, or incubated with rabbit polyclonal antibody against JCPyV VP1 to detect the viral early or late protein, respectively. The primary antibody incubation was followed by incubation with Alexa Fluor 488-conjugated goat anti-mouse or anti-rabbit IgG (Molecular Probes), respectively. Stained coverslips were mounted with antifade fluorescence mounting medium. Two thousand cells per coverslip were counted under a fluorescent microscope (Olympus) for determining the percentage of positive cells.

### 2.11. Real-Time PCR Detection and Relative Quantification

The cDNA was amplified using the specific primer pairs of BKV-VP1 and JCV-VP1 with 2x FAST SYBR Green Master Mix (Topgen Biotech., TW). Nucleotide sequences, 5′-TTGAGTGCTGGGTTCCTGATC-3′ and 5′-GCCCCACACCCTGTTCATC-3′, were used as BKV-VP1 forward and reverse primers. JCV-VP1 forward and reverse primer sequence was 5′-AACAGTGTTGCTTGATGAATTTGG-3′ and 5′-TCTCCACTGCTGGGAACCA-3′, respectively. The PCRs were performed in the StepOnePlus Real-Time PCR system (Applied Biosystems, USA), in accordance with the manufacturer's instructions. The cycling parameters were as follows: (i) 5 min at 95°C; (ii) 40 cycles at 95°C for 3 s and then 60°C for 30 s; and (iii) 95°C for 15 s, then 62°C for 60 s, and then 95°C for 15 s. Specimens were amplified in triplicate with appropriate nontemplate controls. Amplification data were normalized to the human control gene GAPDH expression. Quantification of relative expression was performed using the 2^−ΔΔCt^ relative quantification method.

## 3. Results

### 3.1. Screening Chinese Medicinal Herbal Extracts for Hemagglutination Inhibition Activity on BKPyV VLPs and JCPyV VLPs

The same cell surface molecules that allow binding of BKPyV and JCPyV to host cells are also present on red blood cells, allowing these receptor molecules to mediate erythrocyte recognition and hemagglutination by BKPyV and JCPyV. Enzymatic cleavage of sugar molecules on the cell surface abolished the viruses' ability to hemagglutinate or bind to host cells [20]. Therefore, drugs that can inhibit hemagglutination by VLPs may also be able to block virus binding to cells. In recent years, research on herbal medicines provides a new direction in antiviral drug discovery and development [14], and we wished to identify Chinese medicinal herbs from which inhibitors of BKPyV or JCPyV binding to host cells can be developed. Because the virion and VLP of each polyomavirus recognize the same cellular receptors and VLPs can be obtained more easily and inexpensively, we screened 40 Chinese medicinal herbs for hemagglutination inhibition activity against BKPyV VLPs and JCPyV VLPs. First, substances being screened as potential hemagglutination inhibitors must themselves be free of hemagglutination activity. Of the 40 herbal extracts tested, we found that those of* Carthami Flos*,* Rhodiolae Kirliowii Radix et Rhizoma*,* Artemisia capillaris *Thunb.,* Paeoniae Alba Radix*,* Benincasa hispida*,* Xanthii Fructus*,* Patrinia villosa *Juss.,* Angelicae Dahuricae Radix*,* Isatidis Radix*,* Lycii Fructus*,* Scutellariae Baicalensis Radix*,* Crataegus pinnatifida Fructus*,* Polygoni Multiflori Radix*,* Alismatis Rhizoma*,* Curcumae Wenyujin Radix*, and* Vitis amurensis *did not have detectable hemagglutination activity (Table 1 and see Figure S1A in Supplementary Material available online at https://doi.org/10.1155/2017/5620867). Next, we tested these 16 herbal extracts for the ability to inhibit hemagglutination by BKPyV VLPs (Figure S1B) and JCPyV VLPs (Figure S1C). The results show that only* Rhodiolae Kirliowii Radix et Rhizoma* and* Crataegus pinnatifida Fructus *extracts had substantial hemagglutination inhibition activity. Against BKPyV VLPs, the hemagglutination inhibition titers of* Rhodiolae Kirliowii Radix et Rhizoma* and* Crataegus pinnatifida Fructus *extracts were 2^10^ and 2^14^, respectively (Table 1 and Figure S1B), whereas, against JCPyV VLPs, both titers were 2^12^ (Table 1 and Figure S1C). Thus, of the two herbal extracts,* Crataegus pinnatifida Fructus *has a greater hemagglutination inhibition effect on BKPyV VLPs.

### 3.2. Assessing the Cytotoxicity of* Rhodiolae Kirliowii Radix et Rhizoma* and* Crataegus pinnatifida Fructus *Extracts in HK-2 and SVG-A Cells

**Figure 1:**
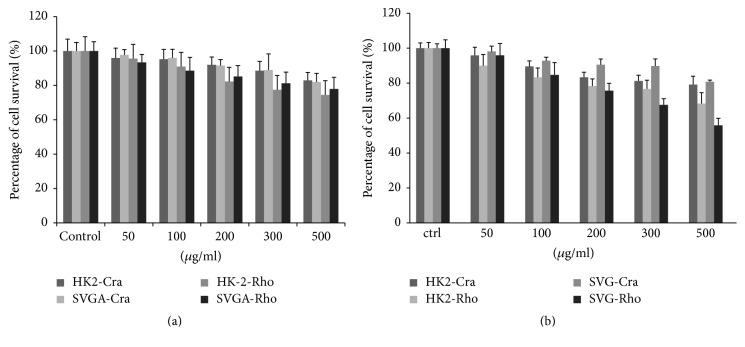
Cytotoxicity of* Rhodiolae Kirliowii Radix et Rhizoma* and* Crataegus pinnatifida Fructus* on HK-2 and SVG-A cells. Cell viability was analyzed using the CCK-8 proliferation assay (a) or trypan blue exclusion test (b) after vehicle or herbal extract treatment for 72 h. Values were normalized to a vehicle-treated control, and three independent experiments were performed and used to calculate standard deviations.

Before testing whether* Rhodiolae Kirliowii Radix et Rhizoma* and* Crataegus pinnatifida Fructus *extracts can inhibit BKPyV and JCPyV infection of host cells, it was important to evaluate the extracts' cytotoxicity on the cells. BKPyV and JCPyV are normally latent in the kidney cells of the human host and reactivate upon immunosuppression. Reactivated BKPyV replicates to high levels in kidney epithelial cells, thereby causing kidney damage in renal transplant patients and resulting in PVAN. Reactivated JCPyV actively replicates in oligodendrocytes in the brain, leading to PML. Accordingly, we chose HK-2 human kidney cells and SVG-A cells, a line of SV40-transformed human fetal glial cells, as the host cells for BKPyV and JCPyV replication, respectively, in our analysis. No marked cytotoxicity was observed in HK-2 and SVG-A cells after treatment with* Rhodiolae Kirliowii Radix et Rhizoma* or* Crataegus pinnatifida Fructus *extract up to a concentration of 300 *μ*g/mL (Figure 1). Furthermore, there is no significant alteration of cell cycle profile distribution under this concentration (data not shown). Therefore, we proceeded to test the two extracts at concentrations below 300 *μ*g/mL to determine their ability to inhibit BKPyV and JCPyV infection of host cells.

### 3.3. *Rhodiolae Kirliowii Radix et Rhizoma* and* Crataegus pinnatifida Fructus* Extracts Inhibit BKPyV and JCPyV Binding to Host Cells

**Figure 2:**
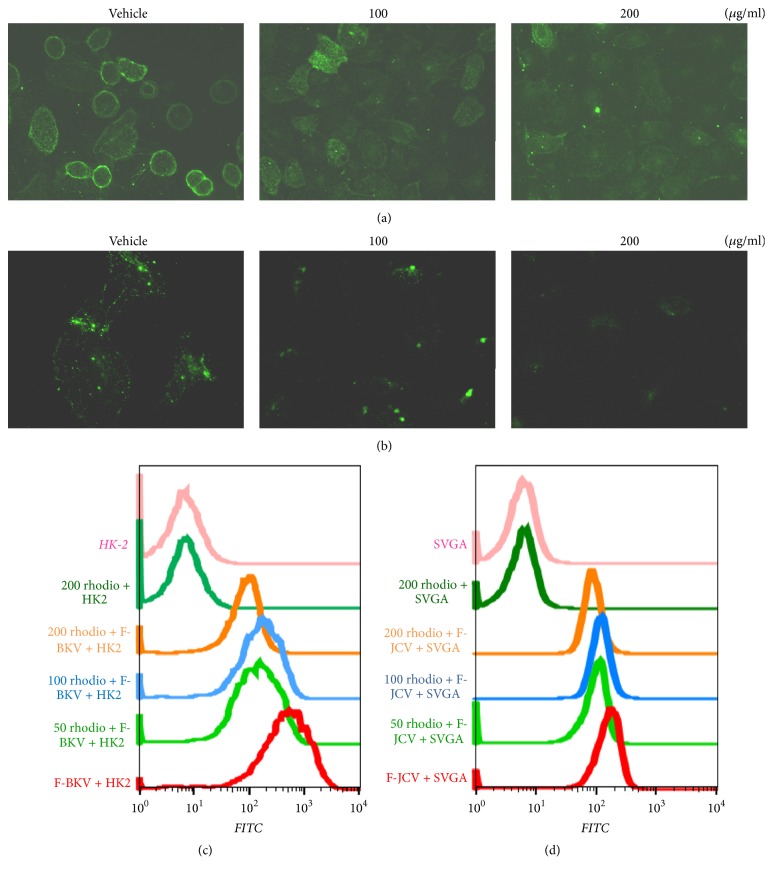
Effect of* Rhodiolae Kirliowii Radix et Rhizoma* treatment on virus binding to cells. (a and b) The effect of* Rhodiolae Kirliowii Radix et Rhizoma *treatment on BKPyV (a) or JCPyV (b) binding to cells was visualized by fluorescence microscopy. (c and d) The inhibition of BKPyV (c) or JCPyV (d) binding to cells by* Rhodiolae Kirliowii Radix et Rhizoma *was analyzed by flow cytometry. Alexa Fluor 488-labeled BKPyV or JCPyV was preincubated with various concentrations of* Rhodiolae Kirliowii Radix et Rhizoma* extract for 1 h at 4°C. The mixtures were then added to prechilled cells and allowed to bind for another 1 h at 4°C. The cells were fixed and washed with ice-cold PBS before analysis by microscopy or flow cytometry. F-BKV, Alexa Fluor 488-labeled BKPyV; F-JCV, Alexa Fluor 488-labeled JCPyV; 50, 100, or 200 rhodio,* Rhodiolae Kirliowii Radix et Rhizoma* used at a concentration of 50, 100, or 200 *μ*g/mL, respectively.

**Figure 3:**
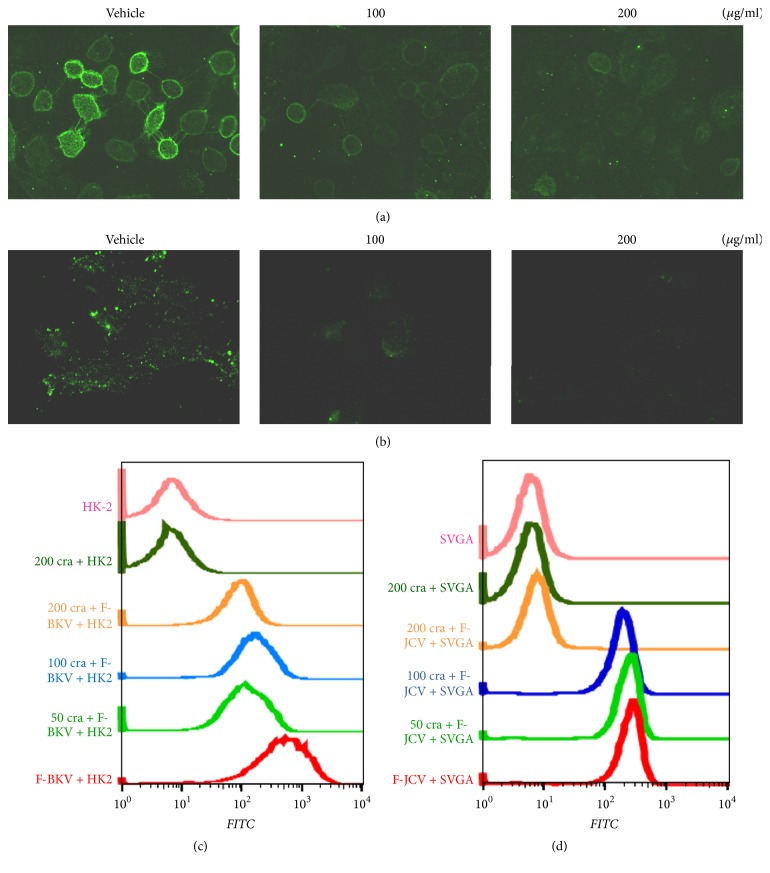
Effect of* Crataegus pinnatifida Fructus* treatment on virus binding to cells. (a and b) The effect of* Crataegus pinnatifida Fructus* treatment on BKPyV (a) or JCPyV (b) binding to cells was visualized by fluorescence microscopy. (c and d) The inhibition of BKPyV (c) or JCPyV (d) binding to cells by* Crataegus pinnatifida Fructus* was analyzed by flow cytometry. Alexa Fluor 488-labeled BKPyV or JCPyV was preincubated with various concentrations of* Crataegus pinnatifida Fructus* extract for 1 h at 4°C. The mixtures were then added to prechilled cells and allowed to bind for another 1 h at 4°C. The cells were fixed and washed with ice-cold PBS before analysis by microscopy or flow cytometry. F-BKV, Alexa Fluor 488-labeled BKPyV; F-JCV, Alexa Fluor 488-labeled JCPyV; 50, 100, or 200 cra,* Crataegus pinnatifida Fructus* used at a concentration of 50, 100, or 200 *μ*g/mL, respectively.

We showed that* Rhodiolae Kirliowii Radix et Rhizoma* and* Crataegus pinnatifida Fructus *extracts have hemagglutination inhibition activity against BKPyV VLPs and JCPyV VLPs (Table 1). We hypothesized that these extracts interfere with the binding of BKPyV and JCPyV to host cells. To visualize an effect on binding, we labeled CsCl-purified BKPyV and JCPyV with FITC. The labeled BKPyV or JCPyV was incubated with different concentrations of* Rhodiolae Kirliowii Radix et Rhizoma* or* Crataegus pinnatifida Fructus *extract, and the resulting mixtures were applied to HK-2 or SVG-A cells. After unbound viruses were washed out, the cells were examined by fluorescence microscopy or analyzed by flow cytometry. Under the microscope, FITC-labeled BKPyV and JCPyV were seen bound to the surface of HK-2 and SVG-A cells, respectively (Figures 2 and 3, vehicle). With increasing concentration of* Rhodiolae Kirliowii Radix et Rhizoma *or* Crataegus pinnatifida Fructus, *we observed a dose-dependent decrease in fluorescence on host cells (Figures 2(a), 2(b), 3(a), and 3(b)). This inhibitory effect was further demonstrated by flow cytometric analysis, in which host cell binding by FITC-labeled BKPyV (Figure 2(c)) and by FITC-labeled JCPyV (Figure 2(d)) was decreased by* Rhodiolae Kirliowii Radix et Rhizoma *in a dose-dependent manner. Similarly,* Crataegus pinnatifida Fructus *caused a dose-dependent decrease in labeled BKPyV's binding to HK-2 cells (Figure 3(c)) and in labeled JCPyV's binding to SVG-A cells (Figure 3(d)). Moreover, at a concentration of 200 *μ*g/mL, treatment with* Crataegus pinnatifida Fructus *extract led to significantly reduced binding of FITC-labeled JCPyV to host cells (Figure 3(d)).

### 3.4. *Rhodiolae Kirliowii Radix et Rhizoma* and* Crataegus pinnatifida Fructus* Extracts Inhibit BKPyV and JCPyV Infection of Host Cells

**Figure 4:**
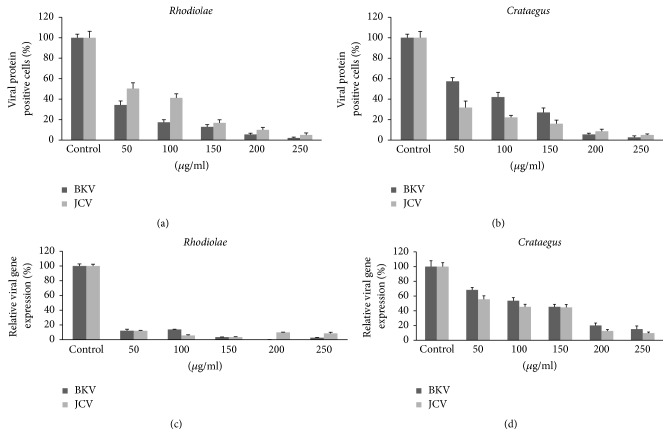
Effect of* Rhodiolae Kirliowii Radix et Rhizoma* and* Crataegus pinnatifida Fructus* treatments on viral protein and gene expression. (a and b) The presence of BKPyV LT or JCPyV VP1 protein was detected by immunofluorescence assay after treatment with* Rhodiolae Kirliowii Radix et Rhizoma* (a) or* Crataegus pinnatifida Fructus* (b) extract. Viral protein expression after treatment with herb extract was calculated as a percentage of the amount observed for the vehicle control, and the results were used for standard deviation. (c and d) BKPyV or JCPyV late VP1 gene expression was determined by quantitative real-time PCR after treatment with* Rhodiolae Kirliowii Radix et Rhizoma* (c) or* Crataegus pinnatifida Fructus* (d) extract. Viral late gene expression after treatment was calculated as a percentage of the amount observed for the vehicle control, and the results were used for standard deviation.

Inhibiting the expression of BKPyV LT resulted in the inhibition of both viral late protein expression and viral genome replication [21]. Thus, LT protein expression serves to indicate BKPyV infection of HK-2 cells. As all SVG-A cells, being SV40 transformed, permanently express SV40 LT, we used expression of the JCPyV major capsid protein VP1 as a marker for JCPyV infection of SVG-A cells. To determine whether* Rhodiolae Kirliowii Radix et Rhizoma *and* Crataegus pinnatifida Fructus *extracts can inhibit infection by BKPyV and JCPyV, we pretreated the host cells, HK-2, and SVG-A with different concentrations of each herbal extract and then infected the cells with BKPyV and JCPyV, respectively. Three days later, immunofluorescence analysis was performed to detect BKPyV LT expression in HK-2 cells and JCPyV VP1 expression in SVG-A cells. The percentage of LT- or VP1-positive cells in infected HK-2 or SVG-A cells, respectively, was significantly reduced by herbal extract treatment (Figures 4(a) and 4(b)). The calculated 50% effective doses against BKPyV and JCPyV were 21.68 and 60.67 *μ*g/mL, respectively, for* Rhodiolae Kirliowii Radix et Rhizoma *(Table 2) and 65.54 and 20.33 *μ*g/mL, respectively, for* Crataegus pinnatifida Fructus* (Table 2). Quantitative real-time PCR was then performed to determine the relative viral late gene VP1 expression after herb extract treatment. As shown in Figures 4(c) and 4(d), BKPyV and JCPyV VP1 gene expression was reduced after treatment of* Rhodiolae Kirliowii Radix et Rhizoma* and* Crataegus pinnatifida Fructus*.

## 4. Discussion

No effective antivirals yet exist for PVAN due to BKPyV or for PML due to JCPyV. In this study, we showed that the extracts of* Rhodiolae Kirliowii Radix et Rhizoma *and* Crataegus pinnatifida Fructus *can inhibit hemagglutination by BKPyV VLPs and JCPyV VLPs. By immunofluorescence and flow cytometry analysis, we further showed that the presence of* Rhodiolae Kirliowii Radix et Rhizoma *or* Crataegus pinnatifida Fructus *extract decreased the binding of fluorescent-conjugated BKPyV and JCPyV to the surface of their respective host cells, indicating that these extracts can effectively interfere with host cell binding by BKPyV and JCPyV. When BKPyV or JCPyV was allowed to infect host cells in the presence of* Rhodiolae Kirliowii Radix et Rhizoma *or* Crataegus pinnatifida Fructus *extract, viral protein expression in the infected cells decreased, and the decrease was progressively larger with increasing concentration of the extract. In addition, the two extracts' inhibitory effects appeared to be specific for the viruses, as neither extract exhibited marked cytotoxicity within the effective concentration range. Collectively, these results support the potential usefulness of* Rhodiolae Kirliowii Radix et Rhizoma *and* Crataegus pinnatifida Fructus* in the development anti-BKPyV and anti-JCPyV drugs.

Virus binding to host cells in the initial stage of the viral life cycle determines the specificity of viral infection and constitutes an important target of antiviral drug research and development. During polyomavirus infection, the VP1 molecule of the viral capsid binds to cellular receptors. Based on the crystallographic X-ray diffraction analysis of murine and simian (SV40) polyomaviruses [22, 23], it is believed that the BC1 and BC2 loops of VP1 form a groove to which sugar moieties of the cellular receptors bind. BKPyV and JCPyV recognize different receptors. The receptor moiety for BKPyV is not yet confirmed; BKPyV is known to recognize N-linked *α*(2,3) sialic acid moieties of a protein and ganglioside molecules GD1b and GT1b. JCPyV uses the serotonergic receptor 5HT_2A_R and *α*(2,6)-linked sialic acid to infect human glial cells. Thus, BKPyV and JCPyV have different specificities for different sugar moieties. Consistently, for both* Rhodiolae Kirliowii Radix et Rhizoma *and* Crataegus Pinnatifida, *we observed different hemagglutination inhibition activities toward BKPyV VLPs and JCPyV VLPs (Table 1).

The anti-BKPyV and anti-JCPyV drugs currently in clinical use lack specificity and have some hepatic, renal, or cardiac toxicity. More antivirals for treating BKPyV and JCPyV infections are in development, and the drug candidates primarily target three stages of the viral life cycle. The first group of such drugs includes chlorpromazine, citalopram, risperidone, ziprasidone, and mirtazapine, which act on the serotonin receptor that binds JCPyV [13, 24], and gallic acid-based compounds, which inhibit the binding of BKPyV and JCPyV to the cell surface [25]. The second group includes retro-2^cycl^ and brefeldin A, which inhibit the retrograde transport of BKPyV and JCPyV to the endoplasmic reticulum [26]. The third group includes cidofovir, cytarabine, ganciclovir, and leflunomide, which work through inhibiting viral replication [27]. Because of the relative ease and low cost of obtaining BKPyV VLPs and JCPyV VLPs, we began by screening medicinal herbs for the ability to inhibit these VLPs' hemagglutination activity before proceeding to further analyses. Although assaying for inhibition of hemagglutination activity was rapid and uncomplicated, substances with intrinsic hemagglutination activity had to be excluded from this assay and thus could not be further tested for inhibition of viral infection. Our preliminary screen resulted in the exclusion of 24 Chinese medicinal herbs on the basis of having hemagglutination activity (Table 1); consequently, we were unable to exclude them as lacking inhibitory activity on virus cell binding. Of the 16 herbs without intrinsic hemagglutination activity, five (*Carthami Flos*,* Paeoniae Alba Radix*,* Isatidis Radix*,* Lycii Fructus*, and* Scutellariae Baicalensis Radix*) did not have a hemagglutination inhibition titer of at least 2^5^, and only* Rhodiolae Kirliowii Radix et Rhizoma* and* Crataegus pinnatifida Fructus* extracts had 2^10^ or higher titers. In fact, we found that the five herbal extracts with hemagglutination inhibition titers < 2^5^ failed to inhibit BKPyV and JCPyV infection of host cells (data not shown). Only* Rhodiolae Kirliowii Radix et Rhizoma *and* Crataegus pinnatifida Fructus* demonstrated inhibition of viral infection. Therefore, a hemagglutination inhibition titer of at least 2^10^ against BKPyV VLPs or JCPyV VLPs may serve as a criterion in future rapid screening tests to indicate drug's potential ability to inhibit cell binding by BKPyV or JCPyV.

We showed that as the dosage of* Rhodiolae Kirliowii Radix et Rhizoma* or* Crataegus pinnatifida Fructus* increased, viral protein expression and virus binding to cells decreased, suggesting that both herbal medicines can block cell infection by BKPyV and JCPyV. We observed potent inhibition of JCPyV cell binding by* Crataegus pinnatifida Fructus*, as evidenced by the nearly abolished binding of fluorescently labeled JCPyV to the surface of SVG-A cells when the JCPyV was preincubated with a high concentration of* Crataegus pinnatifida Fructus *extract (Figure 3(d)). The inhibitory effect of* Crataegus pinnatifida Fructus *on BKPyV was not as pronounced, which is consistent with this herbal extract's IC_50_ values of 65.54 and 20.34 *μ*g/mL on BKPyV and JCPyV, respectively (Table 2). In contrast, the IC_50_ values of* Rhodiolae Kirliowii Radix et Rhizoma* on BKPyV and JCPyV were 21.68 and 60.67 *μ*g/mL, respectively, indicating that* Rhodiolae Kirliowii Radix et Rhizoma* is more effective in inhibiting BKPyV. Yet, even at a high concentration,* Rhodiolae Kirliowii Radix et Rhizoma* was unable to completely block BKPyV binding to cells (Figure 2). In our infection inhibition assays, the herbal extract being tested remained present during the binding of BKPyV or JCPyV to cells at 4°C and was removed along with the virus by washing with PBS only after the binding period. Thus, it is possible that, besides interfering with host cell binding by the viruses, the herbal extracts affected certain other cellular functions, leading to the observed differences in the IC_50_ values for BKPyV and JCPyV. Alternatively, the two herbal extracts' differential inhibitory activities on virus cell binding may stem from the fact that BKPyV and JCPyV recognize different cellular receptors. The present data do not allow us to conclude whether* Rhodiolae Kirliowii Radix et Rhizoma* and* Crataegus pinnatifida Fructus* inhibit BKPyV and JCPyV infection through mechanisms other than inhibition of the viruses' binding to their cell surface receptors.

*Rhodiolae Kirliowii Radix et Rhizoma* and* Crataegus pinnatifida Fructus *are common Chinese medicinal herbs with a long history of therapeutic use.* Rhodiolae Kirliowii Radix et Rhizoma *is reputed to decrease depression, enhance work performance, eliminate fatigue, and prevent high altitude sickness [28], whereas* Crataegus pinnatifida Fructus* has antihyperlipidemic and anti-inflammatory effects [29]. Recent research has also uncovered antiviral activities in* Rhodiolae Kirliowii Radix et Rhizoma* and* Crataegus pinnatifida Fructus*.* Rhodiolae Kirliowii Radix et Rhizoma* was shown to inhibit coxsackievirus B3 infection [30]. Extracted materials from* Crataegus pinnatifida Fructus *exhibited activity against herpes simplex virus [31] and inhibited HIV release from host cells [32]. In the present study, we demonstrated for the first time the antiviral activity of* Rhodiolae Kirliowii Radix et Rhizoma* and* Crataegus pinnatifida Fructus* against human polyomaviruses BKPyV and JCPyV and showed that the activity involves inhibition of BKPyV and JCPyV binding to cells.

Taken together, our results suggest that* Rhodiolae Kirliowii Radix et Rhizoma* and* Crataegus pinnatifida Fructus *are able to inhibit the binding of BKPyV and JCPyV to their cellular receptors.* Rhodiolae Kirliowii Radix et Rhizoma *and* Crataegus pinnatifida Fructus* have long been recognized as natural health products, and their side effects are mild. Although the currently available anti-BKPyV and anti-JCPyV agents have demonstrated effectiveness in in vitro culture systems, many have considerable side effects in a clinical setting. Our findings may lead to future efforts to isolate the effective anti-BKPyV and anti-JCPyV compounds in* Rhodiolae Kirliowii Radix et Rhizoma* and* Crataegus pinnatifida Fructus, *and these potential new drugs may be able to be used in combination with other drugs to achieve an enhanced antiviral effect against BKPyV and JCPyV at lower dosages, thereby reducing side effects.

## 5. Conclusions

The present study showed that the extracts of* Rhodiolae Kirliowii Radix et Rhizoma* and* Crataegus pinnatifida Fructus *inhibit BKPyV and JCPyV infection through interfering with virus binding to host cells. The two herbal medicines' antiviral effects on renal and glial cells may be useful for the treatment of PVAN and PML in the future.

## Supplementary Material

Screening of herbal HI activity on BK and JC VLPs.

## Figures and Tables

**Table 1:** Herbal extracts used in hemagglutination and hemagglutination inhibition screening.

Herb	HA	HAI for BKVLP	HAI for JC VLP
*Carthami Flos* (*紅花*)	−	2^3^	0
*Rhodiolae Kirliowii Radix et Rhizoma* (*紅景*天)	−	2^10^	2^12^
*Artemisia capillaris* Thunb. (*綿茵陳*)	−	0	0
*Polyporus umbellatus* (*豬苓*)	+	ND	ND
*Spatholobi Caulis* (雞血藤)	+	ND	ND
*Atractylodis Macrocephalae Rhizoma* (白朮)	+	ND	ND
*Kaki Calyx* (*柿蒂*)	+	ND	ND
*Artemisia capillaris* Thunb. (*茵陳*)	+	ND	ND
*Paeoniae Alba Radix* (白芍)	−	2^2^	0
*Sparganium stoloniferum* Buch.-Ham. (三稜)	+	ND	ND
*Euonymus laxiflorus* (大*疔黃*)	+	ND	ND
*Polygonati Odorati Rhizoma* (玉竹)	+	ND	ND
*Benincasa hispida* (*冬瓜*子)	−	0	0
*Alpinia oxyphylla* Miq. (*益智仁*)	+	ND	ND
*Xanthii Fructus* (*蒼耳*子)	−	0	0
*Platycodi Radix* (*桔梗*)	+	ND	ND
*Vaccariae Segetalis Semen* (王不留行)	+	ND	ND
*Patrinia villosa* Juss. (*敗醬草*)	−	0	0
*Angelicae Dahuricae Radix* (白芷)	−	0	0
*Bambusae in Taeniam Caulis* (*竹茹*)	+	ND	ND
*Isatidis Radix* (*板藍*根)	−	2^2^	0
*Coptis chinensis* Franchet (*川莫薄*(*川黃連*))	+	ND	ND
*Rehmanniae Radix* (生地黃)	+	ND	ND
*Lycii Fructus* (*枸杞*子)	−	2^2^	2^2^
*Saposhnikoviae Radix* (*防風*)	+	ND	ND
*Scutellariae Baicalensis Radix* (*黃芩*)	−	2^3^	2^5^
*Bu zhong yi qi tang* (補中益氣湯)	+	ND	ND
*Crataegus pinnatifida Fructus* (山楂)	−	2^14^	2^12^
*Polygoni Multiflori Radix* (*首烏*)	−	0	0
*Menthae Haplocalycis Herba* (*薄荷*)	+	ND	ND
*Imperatae Rhizoma* (白茅根)	+	ND	ND
*Alismatis Rhizoma* (*澤瀉*)	−	0	0
*Curcumae Wenyujin Radix* (郁金)	−	0	0
*Curcumae Phaeocaulis Rhizoma* (莪朮)	+	ND	ND
*Polygonati Sibirici Rhizoma* (*黃精*)	+	ND	ND
*Glycyrrhizae Radix* (*甘草*)	+	ND	ND
*Pinelliae Rhizoma* (*半夏*)	+	ND	ND
*Cynanchi Atrati Radix et Rhizoma* (白薇)	+	ND	ND
*Aurantii Submaturus Fructus* (*枳殼*)	+	ND	ND
*Vitis amurensis* (山*葡萄*)	−	0	0

**Table 2:** IC_50_ values of *Rhodiolae Kirliowii Radix et Rhizoma* and *Crataegus pinnatifida Fructus* extracts on BKPyV and JCPyV.

Herb	BKPyV	JCPyV
*Rhodiolae Kirliowii Radix et Rhizoma* (IC_50_, ug/ml)	21.68	60.67
*Crataegus pinnatifida Fructus* (IC_50_, ug/ml)	65.54	20.34

## Abbreviations

BKPyV: BK polyomavirus
JCPyV: JC polyomavirus
PVAN: Polyomavirus-associated nephropathy
PML: Progressive multifocal leukoencephalopathy
PML-IRIS: Progressive multifocal leukoencephalopathy-immune reconstitution inflammatory syndrome
VLP: Virus-like particle
SV40: Simian virus 40
LT: Large T antigen
PBS: Phosphate-buffered saline
CCK-8: Cell Counting Kit 8
HAU: Hemagglutination unit.
