# Supplementary material for: Rhodiolae Kirliowii Radix et Rhizoma and Crataegus pinnatifida Fructus Extracts Effectively Inhibit BK Virus and JC Virus Infection of Host Cells
Source: Evid Based Complement Alternat Med. 2017 Jul 3;2017:5620867. doi: 10.1155/2017/5620867 (PMC5512047; doi:10.1155/2017/5620867)
Supplement: Supplementary file 1 — Screening of herbal HI activity on BK and JC VLPs. [file 5620867.f1.pdf]

Sup. Fig 1

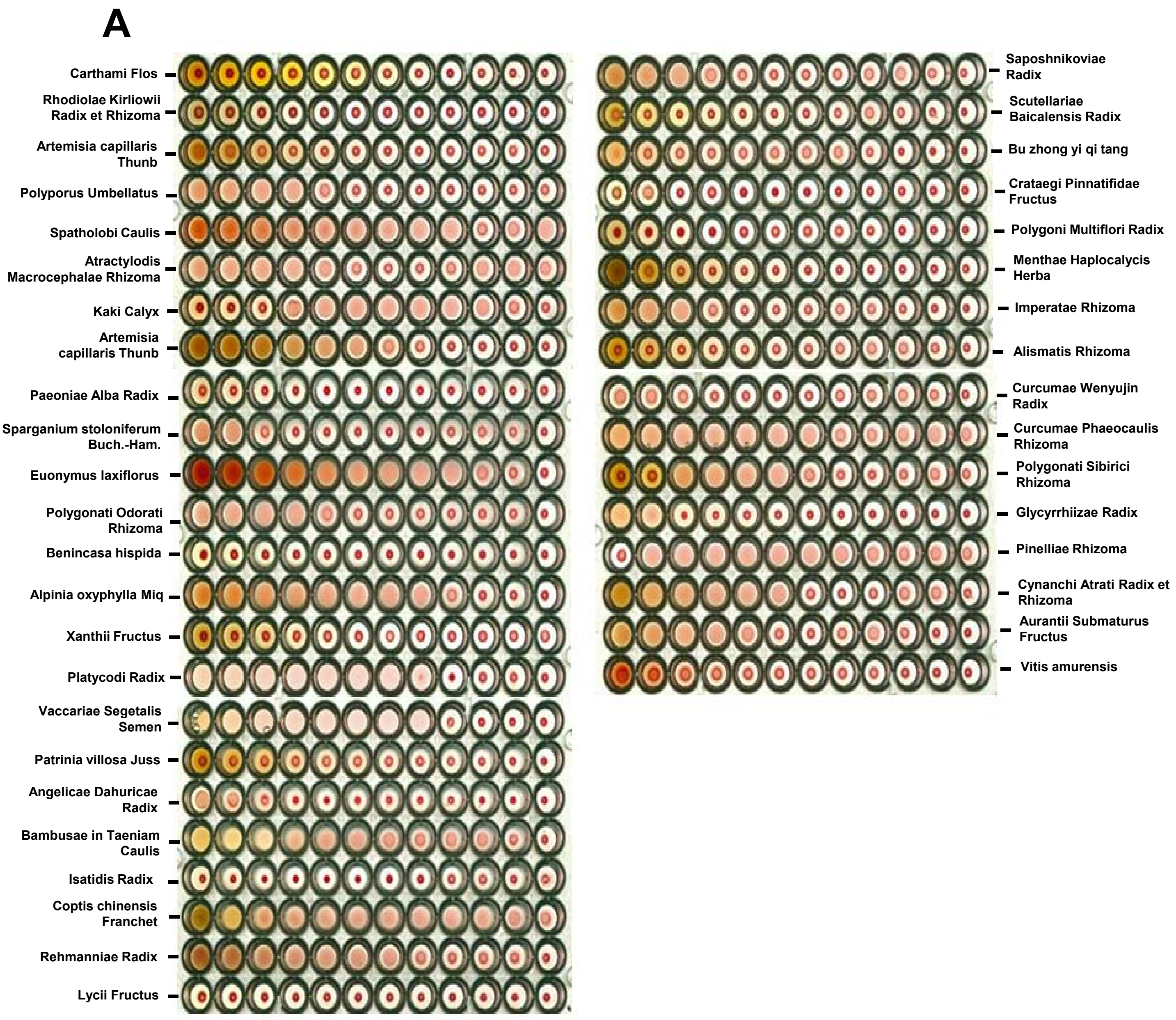

A
Carthami Flos
Rhodiolae Kirliowii Radix et Rhizoma
Artemisia capillaris Thunb
Polyporus Umbellatus
Spatholobi Caulis
Atractylodis Macrocephalae Rhizoma
Kaki Calyx
Artemisia capillaris Thunb
Paeoniae Alba Radix
Sparganium stoloniferum Buch.-Ham.
Euonymus laxiflorus
Polygonati Odorati Rhizoma
Benincasa hispida
Alpinia oxyphylla Miq
Xanthii Fructus
Platycodi Radix
Vaccariae Segetalis Semen
Patrinia villosa Juss
Angelicae Dahuricae Radix
Bambusae in Taeniam Caulis
Isatidis Radix
Coptis chinensis Franchet
Rehmanniae Radix
Lycii Fructus
Saposhnikoviae Radix
Scutellariae Baicalensis Radix
Bu zhong yi qi tang
Crataegi Pinnatifidae Fructus
Polygoni Multiflori Radix
Menthae Haplocalycis Herba
Imperatae Rhizoma
Alismatis Rhizoma
Curcumae Wenyujin Radix
Curcumae Phaeocaulis Rhizoma
Polygonati Sibirici Rhizoma
Glycyrrhiizae Radix
Pinelliae Rhizoma
Cynanchi Atrati Radix et Rhizoma
Aurantii Submaturus Fructus
Vitis amurensis

Sup. Fig 1

B

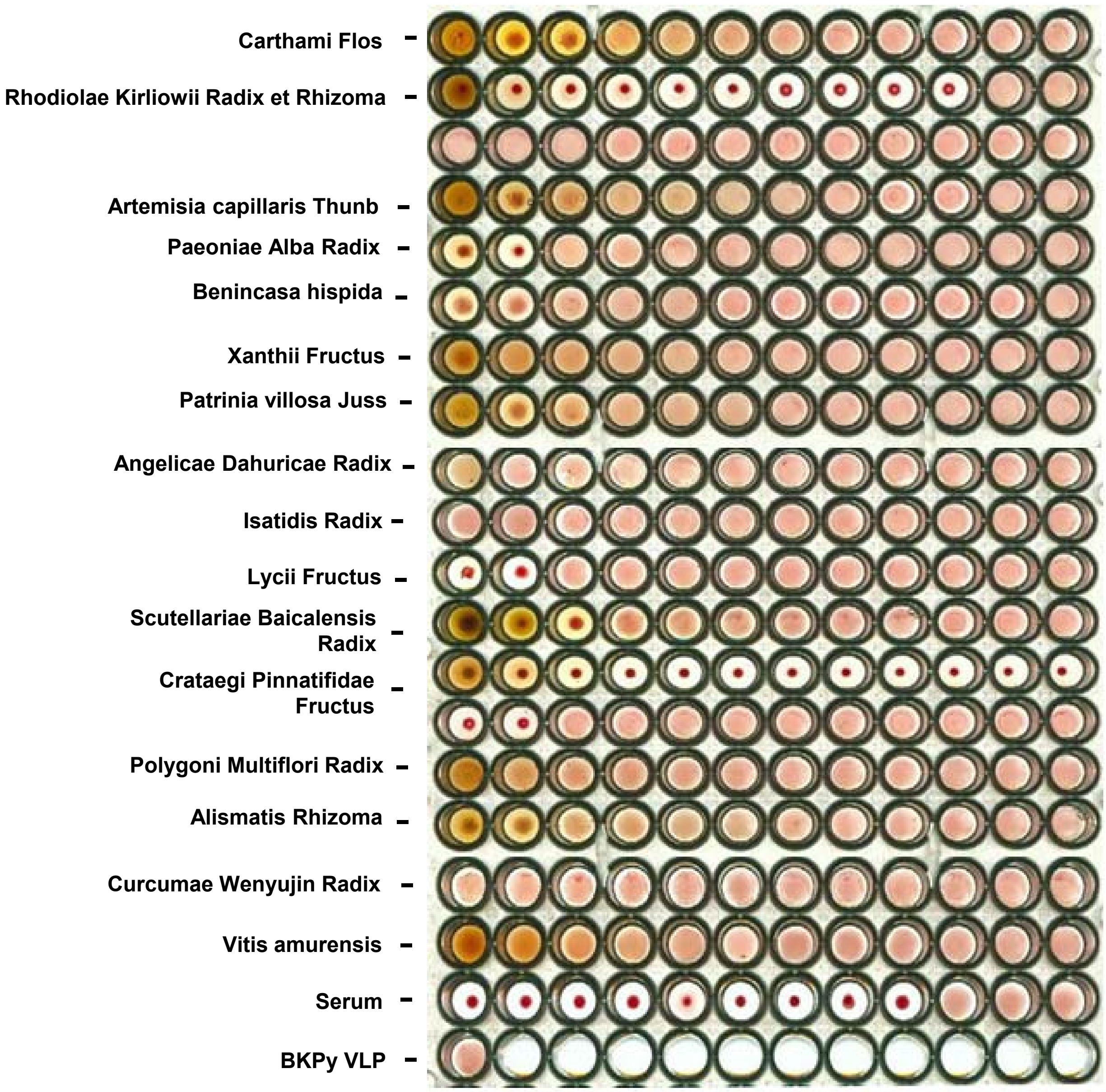

Carthami Flos
Rhodiolae Kirliowii Radix et Rhizoma
Artemisia capillaris Thunb
Paeoniae Alba Radix
Benincasa hispida
Xanthii Fructus
Patrinia villosa Juss
Angelicae Dahuricae Radix
Isatidis Radix
Lycii Fructus
Scutellariae Baicalensis Radix
Crataegi Pinnatifidae Fructus
Polygoni Multiflori Radix
Alismatis Rhizoma
Curcumae Wenyujin Radix
Vitis amurensis
Serum
BKPy VLP

Sup. Fig 1

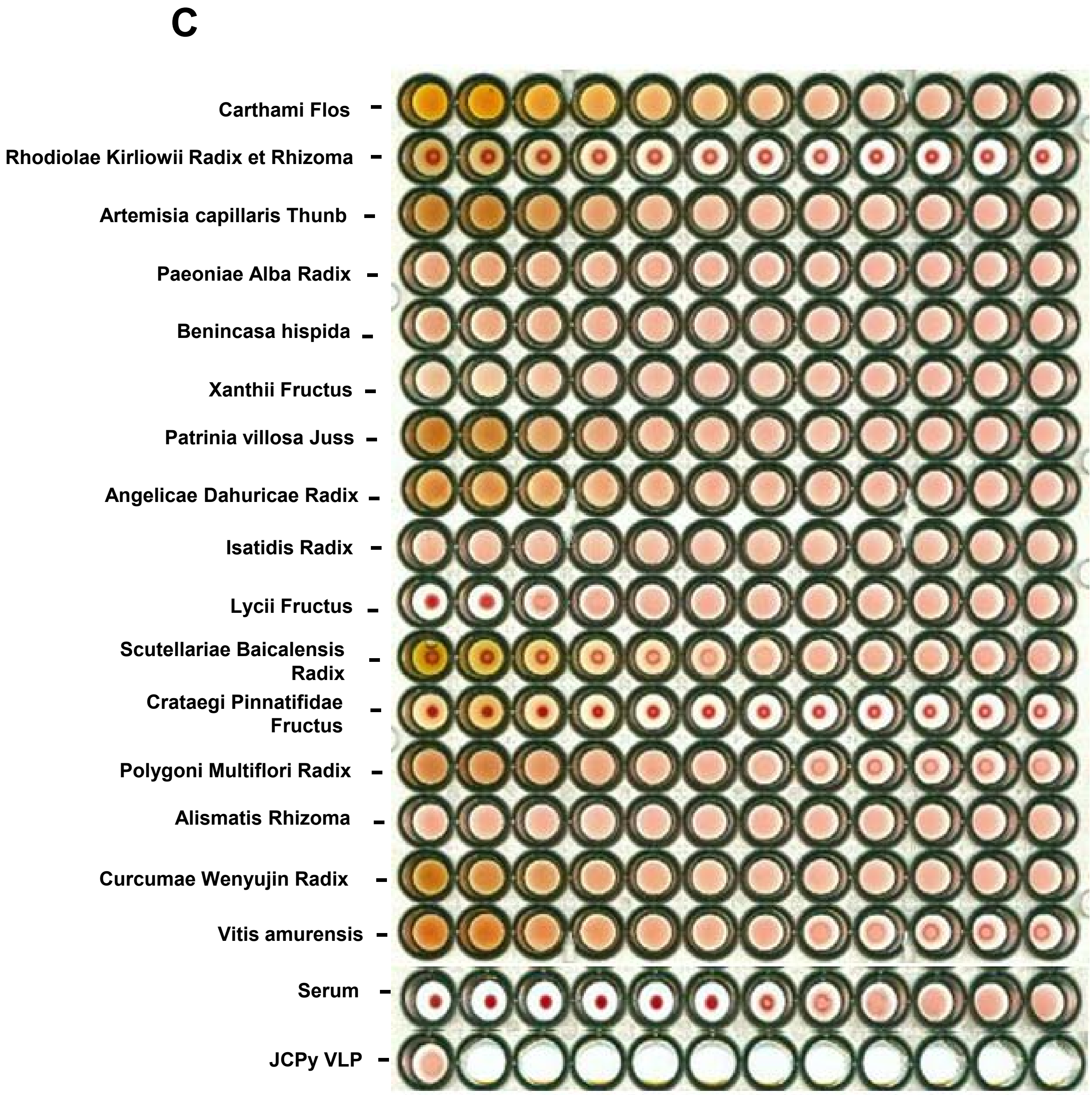
C
Carthami Flos
Rhodiolae Kirliowii Radix et Rhizoma
Artemisia capillaris Thunb
Paeoniae Alba Radix
Benincasa hispida
Xanthii Fructus
Patrinia villosa Juss
Angelicae Dahuricae Radix
Isatidis Radix
Lycii Fructus
Scutellariae Baicalensis Radix
Crataegi Pinnatifidae Fructus
Polygoni Multiflori Radix
Alismatis Rhizoma
Curcumae Wenyujin Radix
Vitis amurensis
Serum
JCPy VLP

Supplementary Fig. S1. Screening of herbal extracts for hemagglutination inhibition activity on BKPyV and JCPyV VLPs. (A) For the hemagglutination assay of herbal extracts, the extracts (100 mg/mL) were diluted in twofold steps with PBS in a U-shaped microtiter plate, 50 μL of 0.7% (v/v) erythrocyte suspension was added to each well, and hemagglutination activity was determined after 1 h incubation. (B and C) For analysis of herbal extracts' hemagglutination inhibition activity on BKPyV VLPs and JCPyV VLPs, the extracts (100 mg/mL) were diluted in twofold steps with PBS in a U-shaped microtiter plate, and 2 HAU of BKPyV VLPs (B) or 8 HAU of JCPyV VLPs (C) in 25 μL were added to each well, followed by 50 μL of 7% (v/v) erythrocyte suspension. After 1 h incubation, the hemagglutination inhibition activity of the extracts was determined.
